# Genome-Wide Identification and Expression Analysis of *GST* Genes during Light-Induced Anthocyanin Biosynthesis in Mango (*Mangifera indica* L.)

**DOI:** 10.3390/plants13192726

**Published:** 2024-09-29

**Authors:** Shiqing Yuan, Chengkun Yang, Bin Zheng, Junbei Ni, Kaibing Zhou, Minjie Qian, Hongxia Wu

**Affiliations:** 1 Sanya Institute of Breeding and Multiplication & Key Laboratory of Quality Regulation of Tropical Horticultural Crop in Hainan Province, School of Tropical Agriculture and Forestry, Hainan University, Haikou 570228, China; 21220951310122@hainanu.edu.cn (S.Y.); hndxyck@hainanu.edu.cn (C.Y.); zkb@hainanu.edu.cn (K.Z.); 2 Key Laboratory of Tropical Fruit Biology, Ministry of Agriculture and Rural Affairs, South Subtropical Crops Research Institute, Chinese Academy of Tropical Agricultural Sciences, Zhanjiang 524013, China; zhengbin@catas.cn; 3 Hainan Institute of Zhejiang University, Sanya 572000, China; nijunbei@zju.edu.cn

**Keywords:** mango, GST, light, anthocyanin, gene expression

## Abstract

Anthocyanins are important secondary metabolites contributing to the red coloration of fruits, the biosynthesis of which is significantly affected by light. Glutathione S-transferases (GSTs) play critical roles in the transport of anthocyanins from the cytosol to the vacuole. Despite their importance, *GST* genes in mango have not been extensively characterized. In this study, 62 mango *GST* genes were identified and further divided into six subfamilies. *MiGSTs* displayed high similarity in their exon/intron structure and motif and domain composition within the same subfamilies. The mango genome harbored eleven pairs of segmental gene duplications and ten sets of tandemly duplicated genes. Orthologous analysis identified twenty-nine, seven, thirty-four, and nineteen pairs of orthologous genes among mango *MiGST* genes and their counterparts in Arabidopsis, rice, citrus, and bayberry, respectively. Tissue-specific expression profiling highlighted tissue-specific expression patterns for *MiGST* genes. RNA-seq and qPCR analyses revealed elevated expression levels of seven *MiGSTs* including *MiDHAR1*, *MiGSTU7*, *MiGSTU13*, *MiGSTU21*, *MiGSTF3*, *MiGSTF8*, and *MiGSTF9* during light-induced anthocyanin accumulation in mango. This study establishes a comprehensive genetic framework of MiGSTs in mango fruit and their potential roles in regulating anthocyanin accumulation, which is helpful in developing *GST*-derived molecular markers and speeding up the process of breeding new red-colored mango cultivars.

## 1. Introduction

Mango (*Mangifera indica* L.) is among the most widely consumed tropical fruits globally, often referred to as the ‘king of tropical fruits’ due to its rich nutritional profile and unique aroma [1]. The coloration of the mango fruit, which varies from green to yellow, blushed red, and full red, is a critical factor influencing consumer choice, with red mangoes being particularly preferred [2]. The red coloration of mango peel results from anthocyanin accumulation, and cyanidin-3-*O*-galactoside is the predominant anthocyanin compound in the mango [3].

Anthocyanins are water-soluble secondary metabolites and play a pivotal role in the growth and development of plants, encompassing a range of functions such as attracting pollinators and seed dispersal animals, conferring resistance to biotic and abiotic stresses, absorbing potentially damaging ultraviolet radiation, acting as antioxidants, and delaying senescence processes [4,5]. Anthocyanin biosynthesis is regulated by environment factors, such as light. High light intensities are known to trigger the accumulation of anthocyanins across a variety of plant species [6]. In addition, the spectral composition of light is also a determinant factor in anthocyanin biosynthesis. Specifically, light with a short wavelength such as blue light and ultraviolet (UV) light has been shown to induce anthocyanin biosynthesis in apple, pear, blueberry, and mango [7,8,9,10,11].

Anthocyanins in plants are generated via a branch of the flavonoid biosynthesis pathway with a set of catalytic enzymes [12,13]. These enzymes comprise phenylalanine ammonia-lyase (PAL), chalcone synthase (CHS), chalcone isomerase (CHI), flavanone 3-hydroxylase (F3H), flavonoid 3′-hydroxylase (F3′H), dihydroflavonol 4-reductase (DFR), anthocyanidin synthase (ANS), and UDP-glucose: flavonoid 3-*O*-glucosyltransferase (UFGT) [12,14,15,16]. After being synthesized in the cytosol, anthocyanins are transported to the vacuole for storage. At present, three theories regarding the transportation of anthocyanin have been proposed: glutathione S-transferase (GST)-mediated transport, membrane transporters, and vesicle trafficking [17].

GST, catalyzing the conjugation of the reduced form of glutathione (GSH) to xenobiotic substrates for the purpose of detoxification, is a large gene family in plants and can be categorized into several subfamilies: Tau, Phi, Lambda, Theta, Zeta, dehydroascorbate reductase (DHAR), tetrachlorohydroquinone dehalogenase (TCHQD), and elongation factor-1 gamma (EF1G) [18]. GST proteins contain two structural domains: an N-terminal conserved reduced glutathione (GSH) binding domain, known as the G-site, and a C-terminal substrate binding domain [19]. The highly conserved N-terminal domain features a typical thioredoxin fold, primarily composed of α-helices and β-strands with a β1-α1-β2-α2-β3-β4-α3 topology, which includes the GSH binding site [20]. The variable C-terminal domain, comprised solely of α-helices, possesses an H-site for the binding of hydrophobic substrates [21]. These two domains are brought into proximity through their three-dimensional structures, forming a catalytic site with specific functions [21]. With the increasing number of whole-genome sequencing projects, GST genes have been identified in numerous species and are known to play critical roles in various aspects of plant growth and development. To date, 79 *GST* genes have been characterized in rice [22], 53 in Arabidopsis [23], 90 in tomato [24], 69 in citrus [25], 25 in apple [26], 67 in sweet cherry [17], and 57 in pear [4].

The function of *GSTs* in facilitating the transport of anthocyanins to the vacuole was first identified in a maize mutant strain, *bronze-2* (bz2, GST-like protein), which exhibited an apparent pigmentation deficit [27]. Subsequent research has unveiled similar roles for *GSTs* in the regulation of anthocyanin levels in diverse plant species. For instance, *LcGST4* in lychee has been associated with the translocation of anthocyanins within the fruit’s pericarp [28]. In the case of grapevines, *VviGST4* plays a crucial role in the accumulation of both anthocyanins and proanthocyanidins (PAs), in contrast to *VviGST3,* which is specifically implicated in PA transport [29]. Furthermore, in apples, the transportation of anthocyanins within the fruit is mediated by *MdGSTF6* [30], and in peaches, *PpGST1* contributes to the vacuolar accumulation of anthocyanins [31]. With the exception of *ZmBz2* from the Tau subfamily, other previously reported anthocyanin-transport-related *GSTs* are clustered in the plant-specific Phi subfamily [32]. Despite the evident involvement of *GSTs* in anthocyanin accumulation in a wide array of species, there is a notable absence of data concerning their specific functions and impact on anthocyanin accumulation in mango fruits.

In this study, mango GST family members were identified from the genome database, and the MiGST subfamily characterization, conserved motifs and domains, and gene structure were analyzed. The expression profiling of *MiGST* genes in different mango tissues and during the process of bagging-, UV-B/white-light-, and blue-light-regulated anthocyanin biosynthesis in mango peel was also conducted by using RNA-seq data and qPCR. Our study provides new insights into the biological roles of MiGST in modulating anthocyanin accumulation in mango.

## 2. Results

### 2.1. Identification, Phylogenetic Analysis, and Physicochemical Properties of GST Family in Mangifera indica

**Figure 1:**
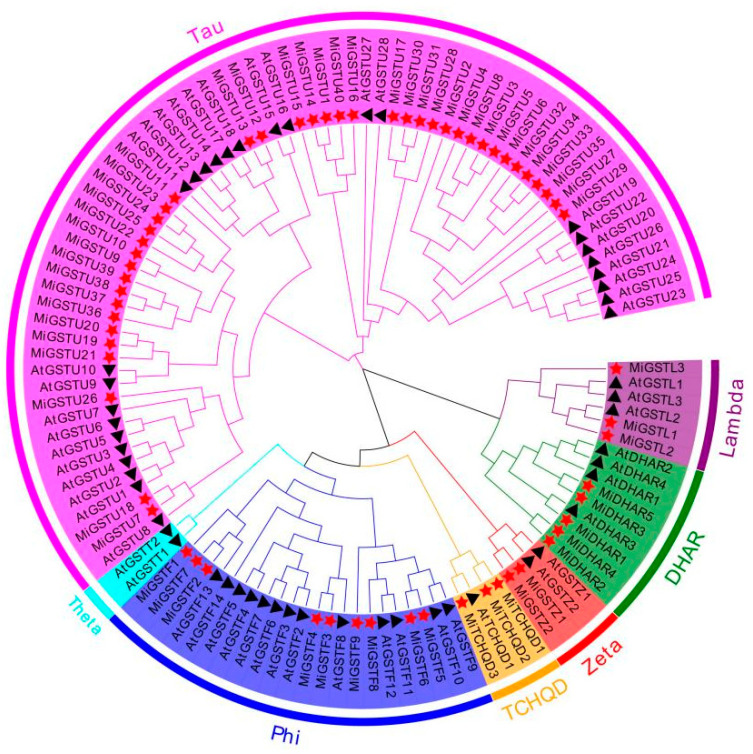
Phylogenetic tree of the characterized MiGST proteins. AtGST proteins were selected as references for representative indicators of each defined subfamily. The *Mangifera indica* (Mi) proteins are marked with red stars, and *Arabidopsis thaliana* (At) proteins are indicated by black triangles, with different subfamilies marked by different colors.

A total number of 62 GST family members were identified from the mango genome database, which were classified into six subfamilies including Tau, Phi, DHAR, TCHQD, Lambda, and Zeta (Figure 1). The Tau subfamily showed the most members with forty proteins (MiGSTU1–MiGSTU40), followed by nine Phi proteins (MiGSTF1–MiGSTF9), five DHAR proteins (MiDHAR1–MiDHAR5), three TCHQD proteins (MiTCHQD1–MiTCHQD3), three Lambda proteins (MiGSTL1–MiGSTL3), and two Zeta proteins (MiGSTZ1–MiGSTZ2) (Figure 1). Notably, no representatives of the Theta subfamily were identified in this comprehensive survey of MiGSTs (Figure 1). The MiGST proteins showed a range of amino acid numbers from 183 (MiGSTU26) to 421 (MiGSTU40), molecular weights from 21.15 kDa (MiGSTU26) to 47.93 kDa (MiGSTU40), and theoretical isoelectric points from 5.04 for MiGSTU26 to 9.25 for MiGSTU40 (Supplementary File S1).

### 2.2. Conserved Motifs and Domains and Gene Structure Analyses of MiGST Proteins

**Figure 2:**
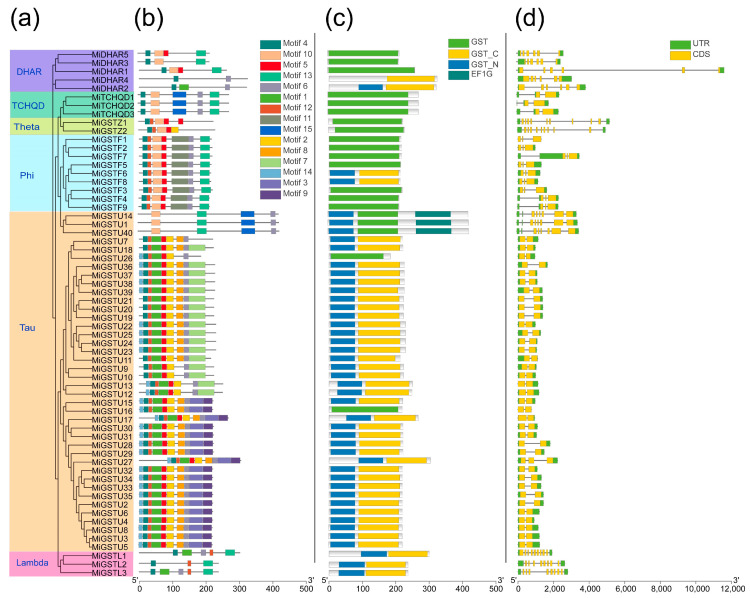
Phylogenetic relationships, conserved motif and domain patterns, and gene structures of mango *GSTs*. (**a**) Phylogenetic tree constructed based on the complete protein sequences of mango GSTs, with different colors corresponding to different subfamilies. (**b**) Display of motifs found in MiGSTs proteins. (**c**) Display of conserved domains found in MiGST proteins. (**d**) Gene structure of *MiGSTs*.

Fifteen conserved motifs were identified in the MiGST proteins by MEME to investigate the conservation and diversity among MiGST family members (Figure 2a,b). The mango GST proteins encompass a variable number of motifs, ranging from two to ten, with motif 4 being present in 59 proteins across all six subfamilies and only absent in MiGSTU1, MiGSTU10, and MiGSTU14 (Figure 2a,b). A notable diversity in motif distribution was detected among the subgroups, and a conserved motif distribution pattern was obtained within individual subgroups (Figure 2a,b). Intriguingly, motifs 3 and 9 were only observed in the Tau subgroup, and motif 11 was exclusive to the Phi subgroup (Figure 2a,b). Conserved domain analysis showed that most GSTs from the Tau and Lambda subfamilies contained GST-C and GST-N domains, and the GST domain was mainly detected in the Phi, DHAR, TCHQD, and Zeta subgroups (Figure 2c). Notably, the EF1G domain was detected in MiGSTU1, MiGSTU14, and MiGSTU40, and MiDHAR4 only contained a GST-C domain (Figure 2c). Gene structure analysis showed that the number of exons in *MiGST* genes spans a range from two to nine, with a notable consistency in exon/intron distribution observed among genes within the same subfamily (Figure 2d). In the Tau subfamily, a majority of the GST members typically contained two exons while, MiGSTU40, MiGSTU14, and MiGSTU1 contained eight exons (Figure 2d). Among the nine plant-specific *GST* genes in the Phi subfamily, six genes (*MiGSTF1/2/3/5/6/8*) contained three exons, and three genes (*MiGSTF4/7/9*) contained four exons (Figure 2d).

### 2.3. Chromosomal Distribution and Syntenic Analysis of MiGST Genes

**Figure 3:**
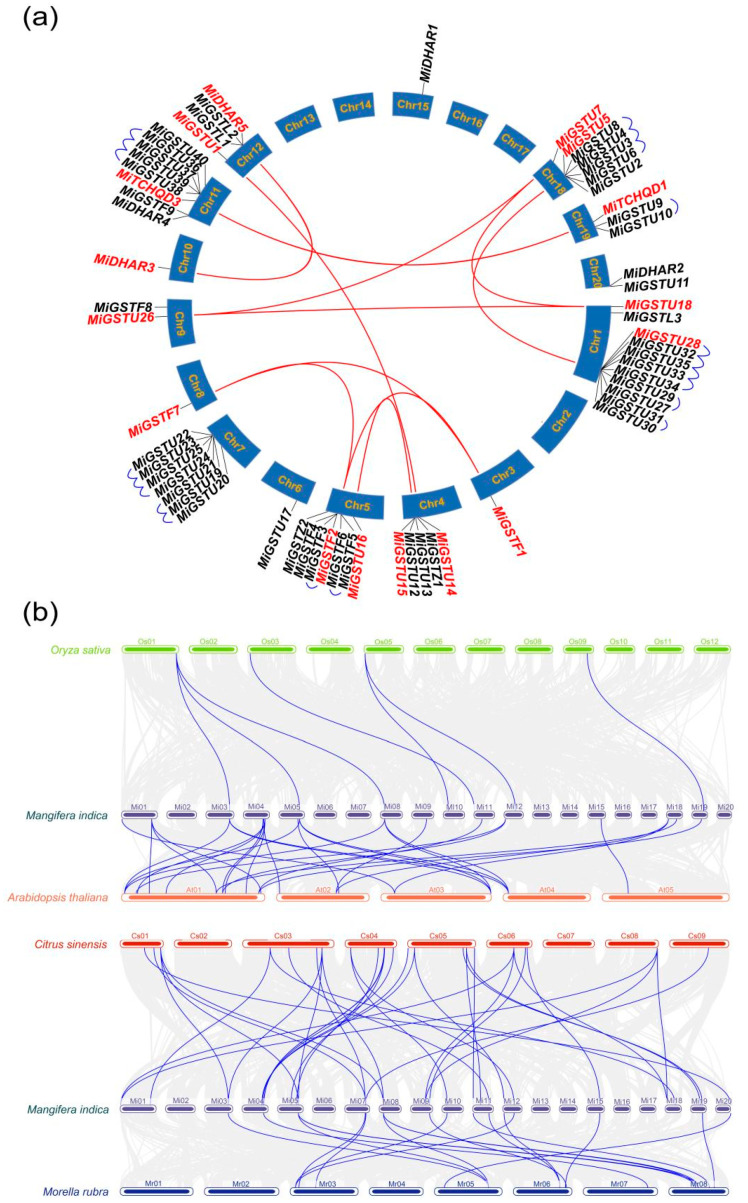
The homology relationships among *GST* genes. (**a**) The mango *MiGST* gene family’s synteny is illustrated, with red lines marking the segmental duplications of *MiGSTs* (highlighted with red color); blue lines indicate the tandem duplications of *MiGSTs*. (**b**) The synteny analysis of *GST* genes across *Mangifera indica*, *Oryza sativa*, *Arabidopsis thaliana*, *Citrus sinensis*, and *Myrica rubra* is displayed, highlighting *GST* gene homologs with purple lines.

The mapping of the 62 *MiGST* genes to the mango genome database revealed that 61 genes are located on 15 chromosomes (Figure 3a). The remaining gene, *MiTCHQD2*, was uniquely mapped to the scaffold NW_025401120.1 due to the incomplete assembly of the mango genome (Supplementary File S1). Of these, the number of *MiGSTs* on the chromosomes from highest to lowest was as follows: chromosome 1 (eleven genes); chromosome 11 (eight genes); chromosomes 5, 7, and 18 (seven genes); chromosome 4 (five genes); chromosome 12 (four genes); chromosome 19 (three genes); chromosomes 9 and 20 (two genes); and chromosomes 3, 6, 8, 10, and 15 (one gene) (Figure 3a). Eleven pairs of segmental gene duplications and ten clusters of tandemly duplicated genes were detected among *MiGSTs* genes (Supplementary File S2, Figure 3a). A synteny analysis of *GSTs* across rice, Arabidopsis, citrus, and bayberry showed that more homologs were detected between mango and the dicotyledonous species, including citrus (thirty-four), Arabidopsis (twenty-nine), and bayberry (nineteen), than between mango and the monocotyledonous rice (seven, Figure 3b).

### 2.4. Tissue-Specific Expression Profiling of MiGST Genes

**Figure 4:**
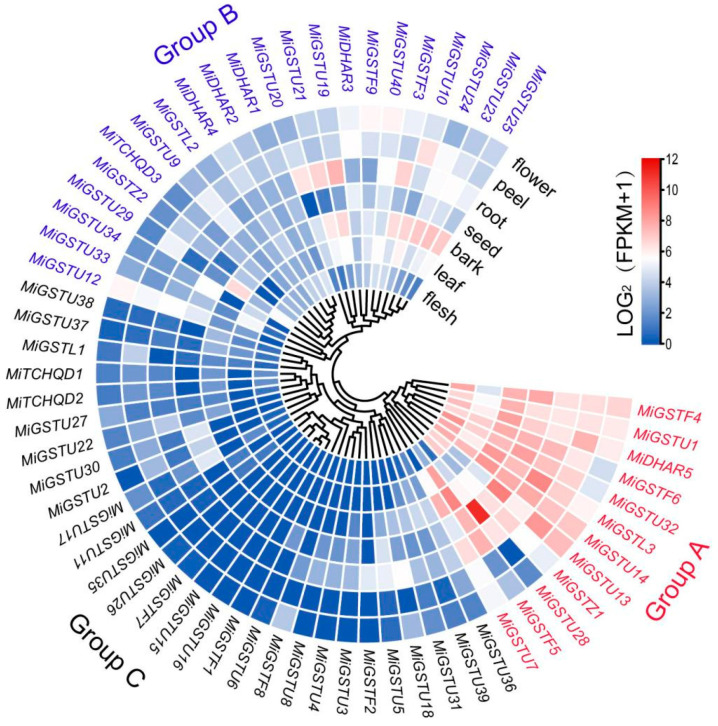
Hierarchical clustering of expression profiles for 62 mango *GSTs* in different tissues. The genes are categorized into Groups A–C. The color bar indicates the expression value from low (blue) to high (red).

Utilizing transcriptome data from the ‘Alphonso’ mango (PRJNA487154), we profiled the tissue-specific expression patterns of 62 *MiGSTs* in mature leaves, bark, seeds, roots, flowers, peel, and flesh. All the *MiGSTs* could be divided into three distinct groups based on their expression profiles: Group A: 12 genes showed high expression levels across the majority of tissues; Group B: 22 genes were highly transcribed in specific tissues; and Group C: 28 genes displayed low expression levels in most tissues (Figure 4). It is noteworthy that the genes with a high expression in the peel included all the genes in Group A and the majority of Group B genes (Figure 4).

### 2.5. Expression of MiGST Genes under Bagging Treatment

**Figure 5:**
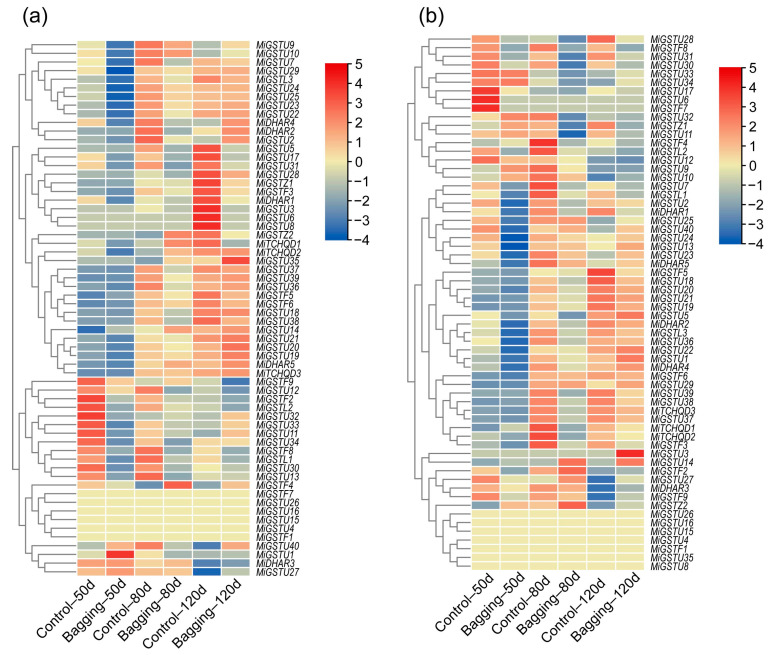
Transcriptional profiling of *MiGST* genes in the peel of ‘Ruby’ (**a**) and ‘Sensation’ (**b**) mangoes under bagging treatment and control conditions for 50, 80, and 120 days after full bloom using RNA-Seq data.

Given the critical influence of light on anthocyanin accumulation in mangoes, ‘Ruby’ and ‘Sensation’ mangoes were treated with double layers of yellow-black paper bags which could block all the sunlight. Our previous study showed that the bagging treatment significantly repressed the anthocyanin accumulation in both ‘Ruby’ and ‘Sensation’ mangoes [3,33]. A comparative transcriptome analysis between bagging-treated and control mangoes which were exposed to sunlight revealed that 10 *MiGST* genes were up-regulated in response to sunlight, including *MiGSTU7*, *MiGSTU13*, *MiGSTU21*, *MiGSTU30*, *MiGSTU31*, *MiDHAR1*, *MiGSTL1*, *MiGSTF3*, *MiGSTF8*, and *MiGSTF9* (Figure 5a,b).

### 2.6. Anthocyanin Concentration and Expression of MiGST Genes under UV-B/White Light and Blue Light Treatments

**Figure 6:**
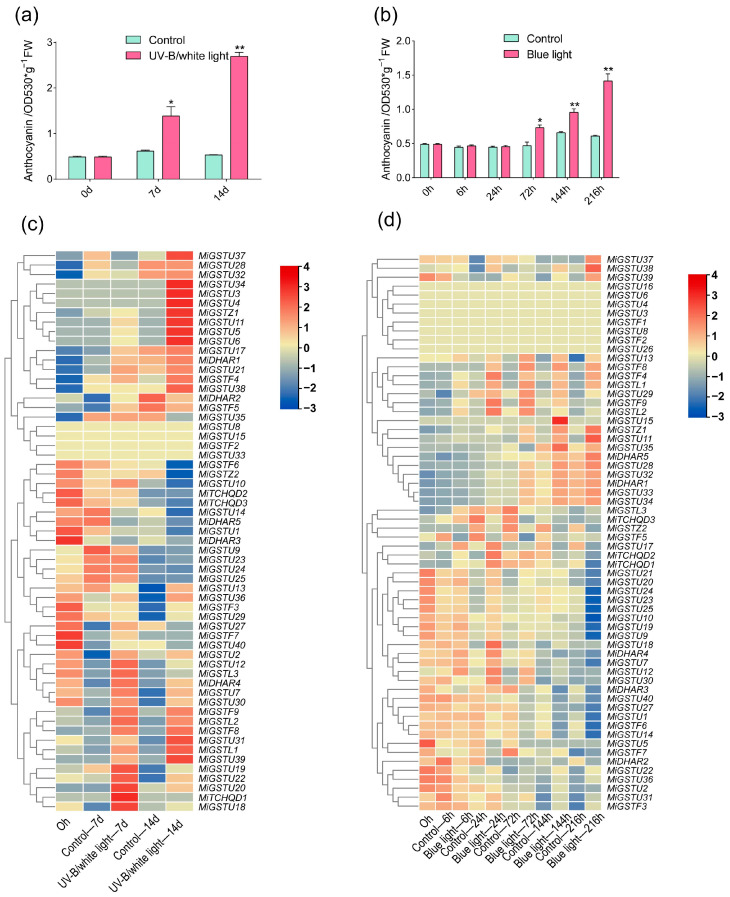
Anthocyanin content in ‘Guifei’ mango peel under UV-B/white light (**a**) and blue light (**b**) treatments. *MiGSTs* expression in ‘Guifei’ mango peel analyzed by RNA-seq under UV-B/white light (**c**) and blue light (**d**) treatments. The data represent the mean ± standard deviation with *n* = 3. * represents significant difference (*p*-value < 0.05); ** represents highly significant difference (*p*-value < 0.01), as determined by Student’s *t*-test.

During UV-B/white light and blue light treatments, the control fruit peel (kept in darkness) showed very low anthocyanin concentrations during the whole treatment, while light-treated fruit peel showed a constantly increasing accumulation pattern (Figure 6a,b). Compared to the control, a significant up-regulation of *MiGSTU18*, *MiGSTU19*, *MiGSTU20*, *MiGSTU22*, and *MiTCHQD1* was observed after a 7-day exposure to UV-B/white light (Figure 6c). In addition, *MiGSTU3*, *MiGSTU4*, *MiGSTU5*, *MiGSTU6*, *MiGSTU11*, *MiGSTU34*, and *MiGSTZ1* showed an up-regulated expression after 14 days of UV-B/white light treatment. Moreover, the expression of *MiDHAR1*, *MiGSTU7*, *MiGSTU13*, *MiGSTU21*, *MiGSTU36*, *MiGSTF4*, *MiGSTF8*, and *MiGSTF9* was markedly induced under UV-B/white light treatment for both 7 and 14 days (Figure 6c). Transcriptome profiling under blue light revealed a significant up-regulation of *MiGSTU7*, *MiGSTU18*, *MiDHAR4*, *MiGSTU12*, and *MiGSTF3* within the initial 6 to 24 h of blue light treatment (Figure 6d). Furthermore, *MiDHAR1*, *MiGSTL1*, *MiGSTF4*, *MiGSTF8*, *MiGSTF9*, *MiGSTU13*, and *MiGSTU29* exhibited substantial increases in expression between 72 and 216 h of blue light treatment (Figure 6d).

### 2.7. The Expression Patterns of MiGSTs during Light-Induced Anthocyanin Accumulation Analyzed by qPCR

**Figure 7:**
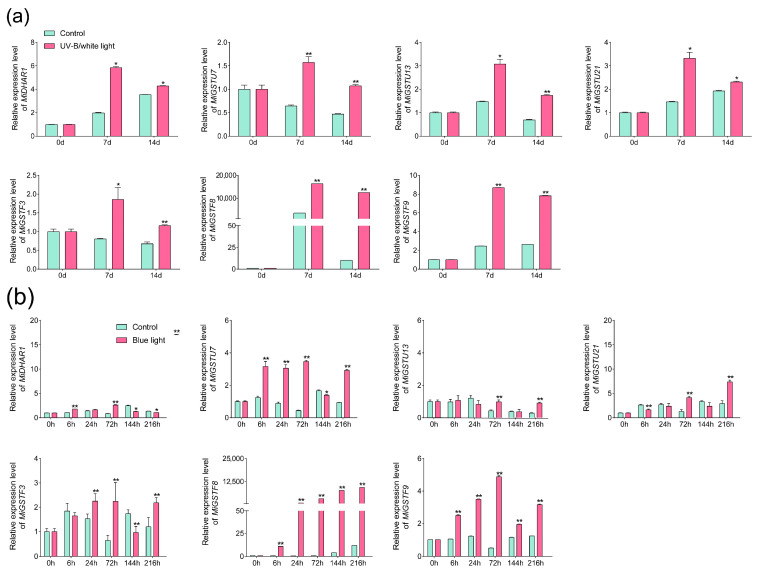
Analysis of *MiGST* genes in ‘Guifei’ mango peel under UV-B/white light (**a**) and blue light (**b**) treatments analyzed by qPCR. Data are depicted as mean values ± standard deviation, derived from three biological replicates (*n* = 3). * indicates a statistically significant difference at *p* < 0.05; ** represents a highly significant difference at *p* < 0.01 between the light-treated and control samples, tested by Student’s *t*-test.

The expression of seven *MiGSTs* which were up-regulated during light-induced anthocyanin accumulation, including *MiDHAR1*, *MiGSTF3*, *MiGSTF8*, *MiGSTF9*, *MiGSTU7*, *MiGSTU13*, and *MiGSTU21*, was also analyzed by qPCR. Under UV-B/white light treatment, all these seven genes were up-regulated, especially *MiGSTF8*, which showed a dramatic up-regulation, with the expression levels in the light-treated mango peel increasing by more than 4 times and 1219 times at day 7 and day 14 compared with control samples, respectively (Figure 7a). The tremendous up-regulation of *MiGSTF8* was also detected under blue light treatment, where its expression was increased by more than 9353 times during the whole treatment, except for at 6 h, when the expression was only up-regulated by 10 times (Figure 7b). *MiGSTU7* and *MiGSTF9* also showed a constant up-regulation under blue light treatment, with the peak of the expression level in blue-light-treated fruit peel at 72 h (Figure 7b). For the remaining four genes, the expression was up-regulated for some time points during blue light treatment, and genes including *MiDHAR1*, *MiGSTU21*, and *MiGSTF3* even showed down-regulation at some time points during the blue light treatment (Figure 7b).

## 3. Discussion

The family of glutathione S-transferase (GST) genes is a rich tapestry of genetic diversity. With the increasing number of genome-sequenced plant species, the whole-genome-wide identification of *GST* genes has been established in diverse plant species, including citrus, Chinese bayberry, apple, pear, etc. [4,25,34,35]. These investigations have shed light on the multifaceted roles of *GSTs* in regulating plants growth and development, protecting plants against biotic and abiotic stresses, modulating secondary metabolism, and participating in signal transduction pathways [18]. In the current research, a total of 62 *GST* genes were identified from the mango genome, which can be further categorized into six subgroups (Figure 1). The number of GST subfamilies differs among plant species. For instance, seven subfamilies of GST were detected in Arabidopsis and citrus, which contain the additional Theta subfamily [25,36]. Apple and tomato contain nine and ten subfamilies, with the additional apple GSTT, GHR, and EF1Bγ subfamilies and tomato Theta, GHR, MGST, and EF1Bγ subfamilies [24,25]. These results indicate that the six subfamilies detected in all plant species show conserved and critical functions among plant species, while the other subfamilies evolve and function in a plant-species-dependent manner. In addition, the Tau and plant-specific Phi subfamilies showed the highest member numbers (Figure 1), which was also observed in apple, tomato, and citrus [24,25,35]. Notably, these two subfamilies have been linked to the critical function of intracellular anthocyanin transport in various fruit species, such as lychee [37], kiwifruit [38], and peach [31].

Gene structure is an indispensable analytical foundation for studying gene evolution and expansion [39]. The analysis of the mango *GST* gene family’s structure revealed that genes within the same group, with similar exon/intron structures and conserved motifs and domains (Figure 2), are likely to share similar functions. Interestingly, some members within the same subfamily exhibit unique motifs, domains, and exon/intron structures, such as *MiGSTU1*, *MiGSTU14*, and *MiGSTU40* in the Tau subfamily, suggesting distinct functions. The majority of *MiGST* genes contain no more than three exons, consistent with previous research in tomato, citrus, and apple [24,25,35], indicating the conserved evolution of the GST family. Whole-genome duplication, segmental duplication, and tandem duplication events are acknowledged as the main drivers behind the generation of new genes and the expansion of gene families [40]. The gene duplication and homology analysis of mango *GST* identified eleven pairs of segmental duplications and ten clusters of tandem duplications (Figure 3a), indicating that both of these processes play a crucial role in the expansion of mango *GSTs*. Comparative collinearity analysis demonstrates a higher degree of collinearity between mango *GST* genes and those of Arabidopsis, bayberry, and citrus compared to rice (Figure 3b), further confirming the mango’s classification as a dicotyledonous plant. Moreover, the highest distribution of homologs was observed between mango and citrus compared to the other species (Figure 3b), suggesting a more recent divergence during the evolution of these two species.

Gene expression patterns are intrinsically linked to their biological roles. The analysis of tissue-specific RNA-seq data in mango has unveiled significant diversity regarding the expression profiles of *MiGST* genes. These genes have been categorized into three clusters based on their relative expression levels. Group A includes 12 genes that are pervasively expressed across the majority of tissues (Figure 4), indicating that these genes function in a wide range of tissues. Group B encompasses 22 genes with tissue-specific expression patterns, and notably, genes such as *MiGSTU10*, *MiGSTU12*, and *MiDHAR3* demonstrated elevated expression levels in the peel (Figure 4), suggesting a crucial role of these genes in regulating the biological process in mango peel. In contrast, Group C consists of 28 genes that are scarcely transcribed in all the analyzed tissues (Figure 4), implying the specialized functions of these genes in other tissues.

Light is an important environmental factor promoting anthocyanin accumulation in fruits, and GST is positively involved in this process. In lychee, the expression of *LcGST4* is up-regulated during debagging-induced anthocyanin accumulation in fruit peel, and the overexpression of *LcGST4* in the Arabidopsis *tt19* mutant lacking an anthocyanin-related *GST* gene promotes anthocyanin biosynthesis in the stem of seedlings [28]. In peach, *PpGST1* expression is highly correlated to the anthocyanin concentration under UVA and UVB treatments, and the overexpression and gene silencing of *PpGST1* significantly increases and decreases anthocyanin accumulation in peach flesh, respectively [31,41]. In this study, the expression of *MiGSTU7*, *MiGSTF8*, and *MiGSTF9* was constantly up-regulated during the light-induced anthocyanin biosynthesis in mango peel (Figure 7a,b), indicating the potential roles of these three *MiGSTs* in regulating anthocyanin accumulation in mango. Interestingly, *MiGSTU7* belongs to the Tau subfamily, and *MiGSTF8* and *MiGSTF9* belong to the Phi subfamily (Figure 1). The Tau and Phi subfamilies are primarily recognized for their role in anthocyanin transport [30,37,38]. These results indicated that GST participating in anthocyanin transportation is conserved during the evolution of plants.

The identification of key genes involved in the process of anthocyanin biosynthesis in fruits is helpful in developing relevant molecular markers, subsequently promoting the breeding of new cultivars. In blood orange, the accumulation of anthocyanin in the flesh is controlled by a *Ruby* gene encoding an MYB TF [42], and PCR-based markers were established for the robust genotyping of *Ruby* locus alleles [43]. As one of the most important TFs regulating anthocyanin biosynthesis, *MYB*-derived molecular markers related to fruit color have been developed in diverse fruit species, including apple [44], grape [45], pear [46], and peach [47]. The white-flower trait in peach is due to a 2 bp insertion or a 5 bp deletion in the third exon of a *GST* gene, and molecular markers based on these variants showed a complete correlation between the *GST* loss-of-function alleles and white flowers in 128 peach accessions [48]. In strawberry, reduced anthocyanin in petioles is caused by a premature stop codon mutation in a *GST* gene, which is a good candidate marker for breeding with the objective of improving fruit color in cultivated strawberry [38]. In the future, sequence variations in the key anthocyanin-biosynthesis-related *GST* genes identified in this study will be analyzed in different-colored mango cultivars, which is helpful in developing *GST*-derived molecular markers and breeding new red-colored mango cultivars.

## 4. Materials and Methods

### 4.1. Whole-Genome Identification of Mangifera indica GST

The mango genome and its associated annotation data (PRJNA487154) were obtained from the National Center for Biotechnology Information (NCBI) [49]. The *Arabidopsis thaliana* GST sequence data were obtained from previous work [25]. Employing the AtGST protein sequence as a reference, we performed a TBtools Blast against the complete mango proteome, followed by the exclusion of any redundant sequences [50]. Subsequently, the Conserved Domain Database (CDD, v3.19; https://www.ncbi.nlm.nih.gov/cdd/ accessed on 11 July 2024) was leveraged to scrutinize conserved structural domains, and only proteins with a complete GST domain were regarded as putative MiGST proteins. ExPASy (https://web.expasy.org/protparam/ accessed on 11 July 2024) was used to analyze the amino acid composition, isoelectric points, and molecular weights of mango MiGST proteins. Furthermore, PSORT (https://www.genscript.com/psort.html accessed on 11 July 2024) was applied to predict the subcellular location of these GST proteins.

### 4.2. Phylogenetic, Gene Structure, and Conserved Motifs and Domains Analyses of MiGST

In this study, 62 GSTs from *Mangifera indica* and 53 GSTs from *Arabidopsis thaliana* (Supplementary File S3) were subjected to multiple sequence alignment using ClustalW 2.0. Subsequently, a phylogenetic tree was constructed using the Maximum Likelihood Method (ML) using the MEGA11 v11.0.8 software [51]. The conserved motifs of the mango MiGST proteins were analyzed using the MEME online tool (https://meme-suite.org/meme/tools/meme accessed on 13 July 2024). Additionally, the gene structure files for the *MiGST* genes were extracted from the mango genome’s GFF annotation file using TBtools-II v2.119 software [50]. TBtools was subsequently utilized to visualize the conserved motifs, the conserved structural domains, and the gene structures of the mango GST proteins [50].

### 4.3. Chromosomal Distribution and Syntenic Analysis

Chromosomal locations for 62 *MiGST* genes were delineated utilizing TBtools [51]. The homology analysis of *GST* genes within *Mangifera indica*, *Arabidopsis thaliana*, *Oryza sativa*, *Myrica rubra*, and *Citrus sinenes* was performed using MCscan X, the plug-in of TBtools-II v2.119 with its default settings [50,52]. The respective genomic sequences for *Arabidopsis thaliana* (PRJNA10719), *Oryza sativa* (PRJNA953663), *Myrica rubra* (PRJNA398601), and *Citrus sinenes* (PRJNA223006) were obtained from the NCBI database.

### 4.4. Plant Material and Treatments

For postharvest UV-B/white light treatment, mature ‘Guifei’ mangoes were harvested from a commercial orchard in the Yazhou District, Sanya City, China, and transported to the laboratory for treatment. Individual fruits were stored in a growth chamber (Bionics, BIC-400, Shanghai, China) and subjected to light treatment, while the remaining fruits were kept as controls in darkness. Each biological replicate consisted of 20 fruits. For UV-B/white light treatment, fruits were subjected to a mixture of 9.0 μW·cm^−2^ UV-B and 40.0 W·m^−2^ white light irradiation, which was generated by two 15 W narrowband UV lamps (SANKYO DENKI, G15T8E, 312 nm, Tokyo, Japan) and ten 30 W light-emitting diode (LED) lamps (NVC, EGZZ1001, Huizhou, China), respectively. The growth chamber was maintained at a temperature of 17 °C and a relative humidity of 80%. At 0, 7, and 14 days of treatment, six fruits were collected from each replicate. The upper fruit peel was removed from the flesh using a peeler, rapidly frozen in liquid nitrogen, and stored at −80 °C for subsequent analysis. The experimental details of ‘Guifei’ mangoes under blue light exposure (453.2 nm, 110 μmol/m^2^/s) are mentioned in a previous study [53].

### 4.5. Acquisition and Analysis of RNA-Seq Data

In this study, four sets of RNA-seq data were obtained from NCBI, including tissue-specific (mature leaves, bark, seeds, roots, flowers, peel, and flesh) transcriptomic data of ‘Alphonso’ mango (*Mangifera indica*, PRJNA487154), the transcriptomic data of ‘Ruby’ and ‘Sensation’ mangoes subjected to bagging treatment (PRJNA905802), and data of ‘Guifei’ mango peel under postharvest UV-B/white light (PRJNA1084921) and blue light treatments (PRJNA854296).

### 4.6. Total Anthocyanin Extraction and Quantification

Anthocyanin concentration measurement was conducted according to our previous study [54]. Briefly, 0.2 g of mango peel tissue was mixed with 1.5 mL of a methanol–hydrochloric acid solution at a ratio of 99:1 (*v*/*v*). The mixture was incubated in darkness at a temperature of 4 °C for 12 h and was subsequently subjected to centrifugation at 12,000 rpm at 4 °C for 10 min. The absorbance of the supernatant was measured at a wavelength of 530 nm using a microplate reader (Nano Quant, infinite M200, Tecan, Männedorf, Switzerland).

### 4.7. RNA Extraction and Gene Expression Analysis

Total RNA was extracted from 0.1 g of frozen mango peel according to the protocol provided with the Pure Plant RNA Preparation Kit (Tiangen, DP441, Beijing, China). The procedures for complementary DNA (cDNA) synthesis and the quantitative polymerase chain reaction (qPCR) were as described by [55]. Primers for qPCR were designed using the Integrated DNA Technologies (IDT) online tool PrimerQuest (https://sg.idtdna.com/pages/tools/primerquest accessed on 18 July 2024) and were normalized to the expression of the mango actin gene. All the primer sequences are listed in the annex (Supplementary File S4). All samples were analyzed in three biological replicates.

### 4.8. Statistical Analysis

The experimental data were subjected to a Student’s *t*-test using SPSS 27.0 (SPSS, Chicago, IL, USA) to analyze the statistical difference between the control and treatment. A *p*-value of <0.05 was considered statistically significant.

## 5. Conclusions

In this study, 62 *GST* genes were identified from the mango genome and classified into six subfamilies, including Tau, Phi, DHAR, TCHQD, Lambda, and Zeta. These genes displayed a high degree of conservation in their exon/intron structures and motif and domain compositions within the corresponding subfamilies. Eleven pairs of segmental duplications and ten clusters of tandem duplications were found among the *MiGSTs*. Tissue-specific expression profiling suggested a significant role of *MiGSTs* in mango growth and development, categorizing the genes into three classes based on their expression levels. Through RNA-seq and qPCR analyses, the significant up-regulation of *MiGSTF8*, *MiGSTF9*, and *MiGSTU7* was observed during light-induced anthocyanin accumulation in mango peel, suggesting their potential involvement in the biosynthesis of anthocyanins in mango. The findings of this study establish a solid foundation for the further investigation of the regulatory role of GSTs in anthocyanin biosynthesis in mango, which is also helpful in developing *GST*-derived molecular markers and breeding new red-colored mango cultivars.

## Data Availability

The data are contained within this manuscript and the Supplementary Materials.

## Supplementary Materials

The following supporting information can be downloaded at https://www.mdpi.com/article/10.3390/plants13192726/s1: File S1: Physicochemical properties of *Mangifera indica* L. GST proteins; File S2: One-to-one orthologous relationship between mango species; File S3: List of the 62 *MiGST* genes identified in this study; File S4: qPCR primers of genes in mango.
